# Running towards amblyopia recovery

**DOI:** 10.1038/s41598-020-69630-7

**Published:** 2020-07-29

**Authors:** Gabriele Sansevero, Claudia Torelli, Raffaele Mazziotti, Alan Consorti, Tommaso Pizzorusso, Nicoletta Berardi, Alessandro Sale

**Affiliations:** 1 Stella Maris Foundation, 56128 Calambrone, Italy; 2 Neuroscience Institute, National Research Council (CNR), via Moruzzi 1, 56100 Pisa, Italy; 3 NEUROFARBA, University of Florence, 50139 Florence, Italy

**Keywords:** Neuroscience, Visual system

## Abstract

Amblyopia is a neurodevelopmental disorder of the visual cortex arising from abnormal visual experience early in life which is a major cause of impaired vision in infants and young children (prevalence around 3.5%). Current treatments such as eye patching are ineffective in a large number of patients, especially when applied after the juvenile critical period. Physical exercise has been recently shown to enhance adult visual cortical plasticity and to promote visual acuity recovery. With the aim to understand the potentialities for translational applications, we investigated the effects of voluntary physical activity on recovery of depth perception in adult amblyopic rats with unrestricted binocular vision; visual acuity recovery was also assessed. We report that three weeks of voluntary physical activity (free running) induced a marked and long-lasting recovery of both depth perception and visual acuity. In the primary visual cortex, ocular dominance recovered both for excitatory and inhibitory cells and was linked to activation of a specific intracortical GABAergic circuit.

## Introduction

In mammals, visual cortex plasticity is maximal during and an early phase of high sensitivity to the external inputs, called critical period (CP), and strongly declines with transition from the juvenile age to adulthood^1–3^. Accordingly, if conditions which cause an early unbalance in the activity of the two eyes (e.g. refractive defects, cataract or strabismus), which lead to alterations in visual cortical development, are not diagnosed and treated before CP end, recovery from these defects is very limited or absent, leading to amblyopia (lazy eye). Amblyopia is a diffused and permanent condition characterized by severe visual deficits, such as reduced visual acuity in the deprived eye and impaired visual depth perception abilities and is a major cause of impaired vision in infants and young children (prevalence 1–5%)^4^.

Increasing evidence supports the possibility to exploit behavioral interventions to enhance plasticity in the primary visual cortex (V1), favoring recovery of visual functions even in adult amblyopic subjects, both in animal models and in humans^1–3,5–11^.

Among the emerging treatments, physical activity appears as particularly promising, due to its remarkable efficacy in animal models and in human subjects^5,10–13^, and for its fully noninvasive nature. Recent findings from independent laboratories demonstrated that physical activity in adult amblyopic rodents promotes visual cortex plasticity, recovery of visual cortical neuron responses to the deprived eye^9^ and recovery from amblyopia^12^; these effects have been linked to the modulation of GABAergic circuitries in V1, a fundamental brake for plasticity in the adult brain^9,12^.

Despite this evidence, several crucial issues remain to be addressed to fully understand the potentialities of physical activity training for translational applications. In particular, nothing is known about the effects elicited by physical activity on visual depth perception abilities, a fundamental issue when considering that the most common deficit associated with amblyopia is an impaired stereoscopic depth perception^14,15^. The recovery paradigms tested so far were based either on a reverse suture approach, not easily applicable to adult human subjects^12^, or involved episodes of physical activity in head-fixed animals^9^ exposed to specific visual stimuli, leaving completely unexplored the possibility to rescue visual functions in adult amblyopic subjects exposed to a totally free running paradigm and conserving binocular sight conditions. Moreover, the long-term maintenance of visual function recovery after the end of physical training, an essential clinical requirement, has not been investigated so far.

Here, we aimed at addressing all these points, taking advantage of a paradigm of voluntary physical activity in adult amblyopic rats with unrestricted access to a running wheel and under ordinary (binocular) viewing conditions.

## Material and methods

### Animal treatment and surgical procedures

All experiments were conducted on Long-Evans black hooded rats. All methods were carried out in accordance with the approved guidelines and regulations of Italian Ministry of Public Health. All experimental protocols were approved by the Italian Ministry of Public Health (approved protocol n. 16C). Animals were housed in a room with a temperature of 21 °C and a 12-h light–dark cycle, with food and water available ad libitum.

### Surgical and experimental procedures

Rats were anesthetized with zolazepam + tiletamine (Zoletil, 1 mg/kg) and monocular deprivation (MD) was performed through eyelid suture at postnatal day (P) 21. Lid margins were trimmed and sutured with 6-0 silk. A post-operative health check control was performed daily until complete cicatrization; subjects with even minimal spontaneous re-opening were excluded. At P70, the long-term deprived eye was re-opened under anesthesia using thin scissors. After reopening of the deprived eye, rats were divided in two groups according to the experimental protocol: sedentary and running animals. A third group of naïve, age-matched animals was added as control group. Sedentary and naïve animals were individually maintained in standard housing conditions, consisting of 40 × 25 × 20 cm cages. Running rats were placed in cages endowed with a running wheel connected to an automatic device recording the number of wheel turns. Animals run an average of 25.20 ± 3.46 km during the first week, 54.30 ± 7.78 km during the second, and 61.81 ± 12.7 km during the third week of treatment.

### Behavioral assessment of visual functions

#### Visual acuity

At the end of the 3 weeks of treatment, RUN (n = 6) and SED (n = 5) rats were subjected to a behavioral assessment of visual acuity. We first measured visual acuity of the open eye (not deprived). Then, we measured visual acuity of the amblyopic eye (by temporary occlusion of the fellow eye) five times, i.e. after eyelid reopening (at P70), immediately at the end of the treatment, and then at 30, 90 and 180 days after the end of the treatment. To measure visual acuity, we used the visual water task^5,16^, which trains animals to first distinguish a low (0.1 cycles deg^−1^) spatial frequency vertical grating from grey, and then tests the limit of this ability at higher spatial frequencies. Once 80% accuracy was achieved, the limit of the discrimination was estimated by increasing the spatial frequency of the grating. Animals were trained 60 trials per day until the achievement of discrimination limit criteria^16^. Visual acuity was calculated as the spatial frequency corresponding to 70% of correct choices on the sigmoidal function fitting the psychometric function. During each session, the experimenter was blind to the experimental group.

#### Visual depth perception

We used n = 7 RUN and n = 8 SED rats. Visual depth perception was assessed as spontaneous exploration in the visual cliff apparatus, as previously described^17,18^. Briefly, the arena was divided into a shallow and a deep side. On the shallow side, a patterned floor was positioned immediately below the glass plate, while on the deep side the checked platform was moved to 29 cm below the glass plate. Each animal was placed on the shallow side, and the total time the rat spent exploring each side of the arena was automatically recorded by the Noldus EthoVision system. The trial ended after 5 min. The arena was cleaned between trials with an alcohol solution. A discrimination index was calculated as follows: (ts − td)/ttot, where ts and td are, respectively, the time spent exploring the shallow side and the deep side of the arena, and ttot is the total time of the test procedure. Each animal was tested only once.

### In vivo electrophysiology

Electrophysiological recordings were performed as previously described^18–20^. Rats were anesthetized with i.p. injection of urethane (1.4 g kg^-1^, i.p., 20% in saline; Sigma-Aldricht) and placed on a stereotaxic frame, with the body temperature maintained at 37 °C. A craniotomy was performed over the binocular visual cortex (4.8–5.2 mm lateral to lambda) and the dura mater was removed. The two eyes were fixed and kept open by means of adjustable metal rings surrounding the external portion of the eye bulb. An electrode (2 × 2-tet-3 mm-150–150-121-A16-15, Neuronexus Technologies) was lowered into the cortex to record local field potentials and single-unit activity. Signals were acquired using a 16 channels Neuralynx device and data analysis was performed using a custom Matlab software. Visual stimuli were generated in Matlab using Psychophysics Toolbox extension and displayed, with gamma correction, on a monitor (Sony Trinitron G500, 60 Hz refresh rate, 32 cd m^−2^ mean luminance) placed 20 cm in front of the animal.

For VEPs, extracellular signal was filtered from 0.3 to 275 Hz and sampled at 30.3 kHz. VEPs in response to square wave patterns with a spatial frequency of 0.03 c/° and abrupt phase inversion (0.5 Hz temporal period), were evaluated in the time domain by measuring the peak-to-baseline amplitude and latency. Computer controlled mechanical shutters were used to alternatively close the two eyes. Visual acuity was obtained by extrapolation to zero amplitude of the linear regression through the data points in a curve where VEP amplitude is plotted against log spatial frequency.

For single-unit recordings, extracellular signal was filtered from 0.6 to 6 kHz and sampled at 30.3 kHz. Spiking events were detected online by voltage threshold crossing and waveforms of 1 ms were acquired around the time of threshold crossing. To improve single unit isolation, recordings from groups of four neighbouring sites (tetrode) were linked, so that each spike was composed by 4 waveforms. Data were loaded on the OffLine Sorter software (Plexon), and a principal component analysis was performed to score spikes with a high degree of similarity in a 3D feature space. Waveforms from each electrode of the tetrodes were processed together to improve isolation. Clusters were progressively defined using convex hulls and then recalculating principal component analysis. Quality of separation was determined based on the following criteria: (1) during manual clusterization with convex hulls, raw waveforms in the clusters were visually inspected to check the uniformity of single waveforms; (2) clusters contained < 0.1% of spikes within a 1.0 ms interspike interval; (3) auto- and cross-correlograms of the clusters were also inspected to reveal if the cluster contained more than a single unit or if several clusters contained spikes of the same unit; (4) the peak amplitude of a unit remained stable over the entire recording session. Units were included in the sample for analysis of tuning properties when they had an average peak firing rate, across trials of the optimal stimulus for the dominant eye, of > 0.5 Hz. Drifting sinusoidal gratings were used as visual stimuli (1.5 s duration, temporal frequency of 2 Hz, 12 different orientations with a step of 30°, 3 spatial frequency: 0.02, 0.04, 0.08 c/°). Stimulation was repeated five times per eye, with stimulus conditions randomly interleaved, and two grey blank conditions (mean luminance) were included in all stimulus sets to estimate the spontaneous firing rate. The average spontaneous rate for each unit was calculated by averaging the rate over all blank condition presentations. Responses at each orientation and spatial frequency were calculated by averaging the spike rate during the 1.5 s presentation and subtracting the spontaneous rate. The preferred stimulus was determined finding the combination of spatial frequency and orientation that maximize the response, independently for each eye. Orientation tuning curves were constructed for the spatial frequency that gave maximal response at this orientation. Given this fixed preferred orientation (OPref), the tuning curve was fitted as the sum of two Gaussians centred on OPref and OPref + π, of different amplitudes but equal width, with a constant baseline. From this fit, we calculated two metrics: an OSI representing the ratio of the tuned versus untuned component of the response, and the width of the tuned component. OSI was calculated as follows: (respOPref-respOOrtho)/(respOPref + respOOrtho), where resp is the maximal response evoked by visual stimulation and OOrtho is the orientation orthogonal to the preferred one. Tuning width is the half-width at half-maximum of the principal gaussian. In addition, DSI was calculated as: (respOPref-respOOppo)/(respPref + respOppo). ODI was calculated as follows: ODI = (respContra-respIpsi)/(respContra + respIpsi), where Contra is the contralateral eye and ipsi is the ipsilateral eye. Narrow spiking and broad spiking units distinctions was performed by analyzing properties of their average waveform: the height of the positive peak relative to the initial negative trough, the time from the minimum of the initial trough to maximum of the following peak, and the slope of the waveform 0.5 ms after the initial trough. Two linearly separable clusters were found, corresponding to narrow-spiking (putative inhibitory) and broad-spiking (putative excitatory) neurons. Both k-means and linkage clustering identically separated these clusters.

### Immunohistochemistry

After the period of differential rearing, rats were subjected to eyelid suture of the fellow eye, placed in a dark light-proof room for two days, and then re-exposed to light for 2 h (with this protocol, visual stimulation was maximal for the previously deprived eye). Rats (n = 6 naïve, n = 6 SED, and n = 5 RUN) were anesthetized with an overdose of chloral hydrate (10% in saline; Sigma Aldrich) and perfused transcardially with Phosphate Buffered Saline (10 mM; PBS) followed by fixative (4% Paraformaldehyde, 0.1 M Sodium Phosphate, pH 7.4). The brain was then removed and post-fixed for 3 h in the same fixative (4%) at room temperature (RT), before being washed three times with PBS and immersed in 30% sucrose until complete precipitation. Brain slices of 50 µm thickness were cut with a sliding microtome (Leitz, Germany). All reactions were performed on free-floating sections. After a blocking step in 10% NGS (Normal Goat Serum) and 0,4% Triton X-100 in PBS (1 h at RT) a double staining for C-Fos and GABAergic inhibitory interneurons markers was done used a primary antibodies’ solution with 2% NGS, 0,2% Triton X-100, 1:1000 guinea pig anti C-Fos polyclonal antibody (Synaptic System, Gottingen, Germany), and either 1:2000 mouse anti-Parvalbumin, 1:2000 (Sigma Aldrich), rabbit anti-Somatostatin (DBA Italia s.r.l., Italy), or 1:500 rabbit anti-Vasoactive Intestinal Peptide (DBA Italia s.r.l., Italy), in PBS (overnight at 4 °C). Antigen–antibody binding was revealed with a solution composed by 1% NGS, 0,1% Triton X-100, 1:500 goat Alexa Fluor 448 anti-guinea pig and 1:500 goat Alexa Fluor 568 anti-rabbit (2 h covered at RT). Sections of the three experimental groups were reacted together with the same immunohistochemical procedure. Sections were acquired using a high resolution fluorescent microscope (Zeiss, Germany) to analyze the number of active GABAergic-positive interneurons. Images were imported to Fiji ImageJ Software^21,22^ and counts were done in blind during the entire analysis. Slices were mounted on glass slides and covered with VectaShield mounting medium (Vector Labs, USA). Images from the binocular primary visual cortex were acquired at 20× magnification. For each animal, 5 to 7 slices were acquired and, for each section, labeled cells were counted in three different sample windows, corresponding to layers 2/3, layer 4 and layers 5/6, in the V1 area. Double stained cells were counted manually using a blind procedure, and their density was calculated using Fiji ImageJ Software^21,22^. The obtained values, expressed as cells/mm^2^, were used for statistical analysis. Since no significant activation of cells was found in layer 4, the analysis was focused on layers 2/3 and layers 5/6.

### Experimental design and statistical analyses

Statistical analysis was done using SigmaStat Software. Data were tested for normality before running statistical tests; parametric tests were run on normally distributed data and, in case normality test failed, non-parametric tests were performed as appropriate. Differences between two independent groups were assessed with a two-tailed t-test; differences between two dependent groups (e.g. visual acuity of the deprived and fellow eyes in the same animals) were assessed with a two-tailed paired t-test. One-way ANOVA, Two-way ANOVA and Two-way RM ANOVA, followed by Holm–Sidak multiple comparison procedure, were used to compare normally distributed data belonging to more groups. One-way ANOVA on ranks, followed by Dunn’s method or Tukey test, were performed to compare not normally distributed data belonging to more than two groups. Level of significance was *p* < 0.05, unless otherwise specified. The size of biological replicates is indicated by the n numbers in the various experimental sections. The two sample Kolmogorov–Smirnov test was used to compare two different distributions.

## Results

### Voluntary physical activity induces long-term recovery of visual functions

**Figure 1:**
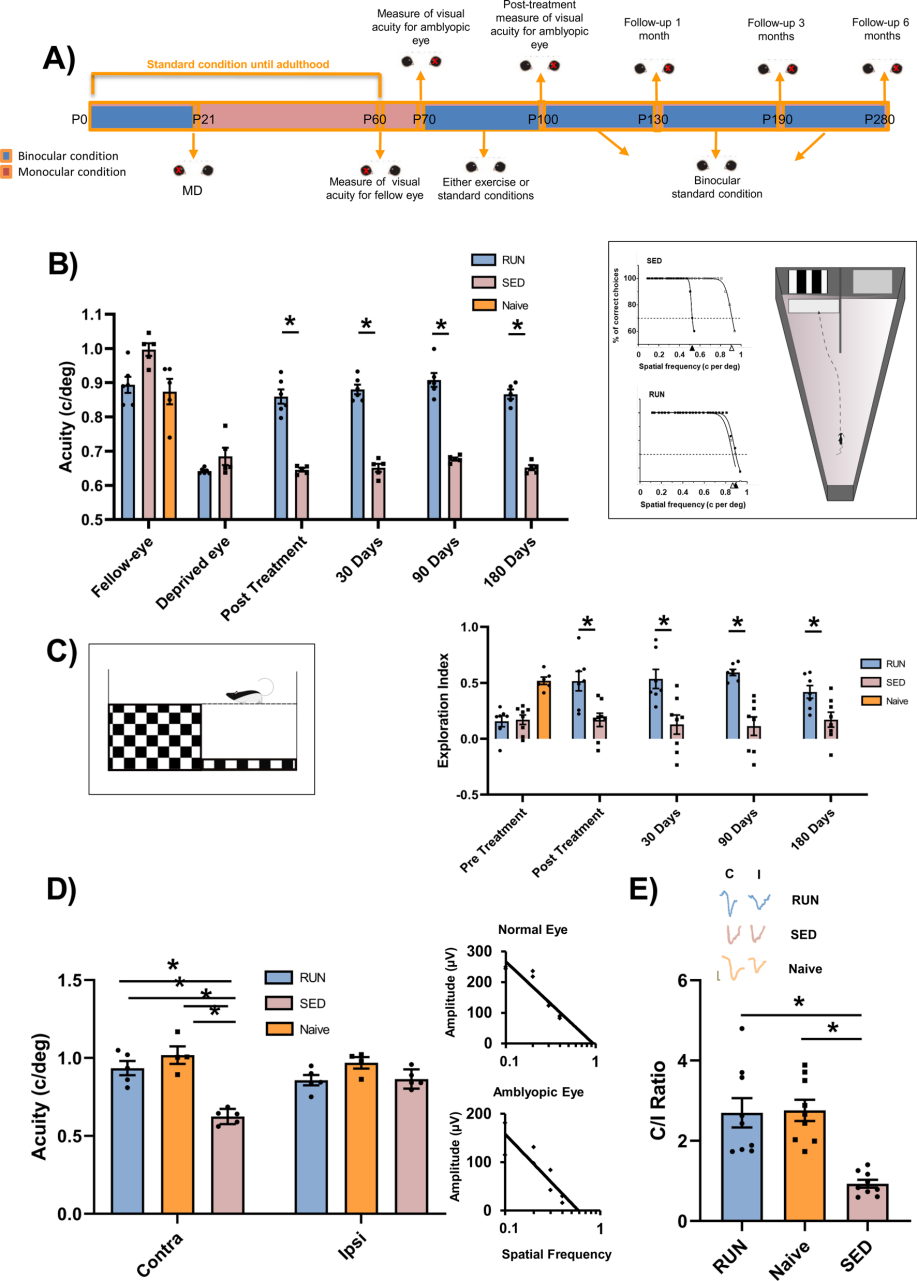
Voluntary physical activity induces long-term recovery of visual functions. (**A**) Schematic diagram of the protocol. (**B**) Visual acuity through the long-term deprived and the fellow eye was measured using the visual water box task (right, inset). At the end of the physical activity period, visual acuity of the previously deprived eye was different from that of the fellow eye in SED rats (Two Way RM ANOVA, *p* < 0.001), but not in RUN animals (*p* = 0.403). Two-Way RM ANOVA revealed that, in RUN rats, the visual acuity of the previously deprived eye measured immediately after the end of the three weeks of physical activity was significant increased with respect to that measured before treatment (p < 0.001) and remained unaltered 30, 90 and 180 days after the end of the treatment (p = 0.760, *p* = 0.178 and *p* = 0.996, respectively). In contrast, the visual acuity of the long-term deprived eye did not change throughout the study in SED rats (Two-Way RM ANOVA with Holm-Sidak method, pre-treatment vs. post-treatment, *p* = 0.403; pre-treatment vs. 1 month after treatment, *p* = 0.575; pre-treatment vs 3 months, *p* = 0.992 and pre-treatment vs 6 months, *p* = 0.566). (**C**) Visual depth perception was assessed using the visual cliff task (left, inset), as the exploration preference for the shallow and depth side of the arena. One-way ANOVA showed a significant preference for the shallow side in RUN animals, which exhibited an exploration index statistically higher, respectively, than that of SED rats (Holm-Sidak method, *p* < 0.05). All animals were tested after restoration of binocular vision. (**D**) Electrophysiological recordings of visual evoked potentials form the primary visual cortex. In SED rats, visual acuity of the deprived eye remained significantly lower with respect to the other eye (One Way RM ANOVA, Holm-Sidak method, *p* < 0.001); in contrast, a full visual acuity recovery was achieved by RUN rats (*p* = 0.101) that showed values not different from those of naïve animals (*p* = 0.127). (**E**) Ocular dominance was assessed through the C/I VEP ratio in response to a low spatial frequency grating. The C/I VEP ratio was significantly higher in RUN than in SED rats (One-way ANOVA on ranks, Tukey Test, *p* < 0.05), but not different from that of naïve rats (n = 9; *p* > 0.05). Error bars indicate s.e.m.; * indicates statistical significance.

To investigate the effects of voluntary physical activity on visual function recovery in adult amblyopic rats, we compared visual abilities in a group of adult long-term deprived rats subjected to three weeks of completely free running (in cages endowed with a running wheel), with those of sedentary animals reared in conventional standard conditions, and with those of non-deprived (naïve) rats. The experimental protocol is depicted in Fig. 1A.

We first performed a behavioral measure of visual acuity through the visual water-box task^5,16^. Rats were conditioned to distinguish a low spatial frequency grating from the gray, with high reliability (training phase). Each animal’s ability limit was then assessed at higher spatial frequencies. This allowed us to follow visual acuity longitudinally in the same individuals, measuring spatial discrimination abilities through the previously deprived eye five times, i.e. immediately before treatment, immediately at the end of physical training, and at three additional follow-ups (1, 3, and 6 months past the end of the treatment). After three weeks of running, a full recovery of visual acuity was evident in exercised animals (RUN rats, n = 6) (amblyopic eye: 0.86 ± 0.02 c/deg; fellow eye: 0.89 ± 0.02 c/deg); visual acuity recovery was instead totally absent in control sedentary rats (SED rats, n = 5; amblyopic eye: 0.64 ± 0.01 c/deg; fellow eye: 0.99 ± 0.02 c/deg) (Two-Way RM ANOVA, treatment × time F = 39.078, DF = 4, Holm-Sidak method, *p* = 0.403 for RUN rats, *p* < 0.001 for SED rats) (Fig. 1B).

In the follow up assessments, visual acuity recovery in RUN rats turned out be maintained at all time points after the end of the exercise treatment (Two-Way RM ANOVA, Holm-Sidak method, post-treatment vs. 1 month after treatment, *p* = 0.760; post-treatment vs. 3 months, *p* = 0.178; post-treatment vs. 6 months, *p* = 0.996) (Fig. 1B). At the end of the treatment, visual acuity measured in the formerly amblyopic eye of RUN animals was equal to visual acuity of Naïve animals, and both were higher than visual acuity of SED rats at the same time-point (Naïve, 0.87 ± 0.03 c/deg; One Way ANOVA, F = 25.319, DF = 2, Holm-Sidak method, *p* = 0.838, *p* < 0.001, *p* < 0.001, respectively).

Next, we investigated whether physical activity affects visual depth perception abilities in the visual cliff task. This test exploits the spontaneous tendency of rodents to avoid the deep side of a visual cliff arena (Fig. 1C). To discriminate the deep from the shallow side, binocular vision is needed: rats under monocular vision condition (one eye acutely occluded) display no preference for the shallow side of the arena, while rats with binocular vision do^17^. Amblyopic rats are severely impaired in the visual cliff task, displaying no preference for the shallow side of the arena, with no significant difference with respect to rats under monocular conditions^17^.

We found no preference for the shallow side of the arena in SED rats (n = 8; exploration index = 0.16 ± 0.06), while RUN rats (n = 7) displayed a clear preference for the shallow side, with an exploration index (0.51 ± 0.08), significantly higher than that of SED rats (Fig. 1C) and equal to that of naïve rats with normal binocular vision (n = 6; 0.520 ± 0.03) (One-way ANOVA, between groups F = 9.717, DF = 2, Holm-Sidak method, *p* < 0.05, *p* = 0.977, respectively). Strikingly, rescue of visual depth perception abilities in RUN rats was long lasting, persisting for the entire 6-month follow-up period (Fig. 1C). (Two-way repeated measure ANOVA, F = 30.638, DF = 4, Holm-Sidak method, time among RUN, pre-treatment vs 1 month, *p* = 0.001; pre-treatment vs 3 months, *p* < 0.001; pre-treatment vs 6 months, *p* = 0.045).

To assess whether the recovery of vision evident at the behavioral level was related to changes in visual responses in the primary visual cortex, we performed, in a separate group of animals, electrophysiological recordings from V1; visual acuity and ocular dominance were assessed by means of visual evoked potentials (VEPs) from V1. Recordings occurred immediately after the end of the physical activity period. In SED rats (n = 5), visual acuity of the deprived eye remained significantly lower (0.62 ± 0.02 c/deg) with respect to the other eye (0.86 ± 0.03 c/deg) (Two-Way RM ANOVA, Holm-Sidak method, F = 25.601, DF = 2, *p* < 0.001) (Fig. 1D). In contrast, we found no difference between the visual acuity values of the two eyes in both RUN rats (n = 5, controlateral visual acuity: 0.93 ± 0.04 c/deg; ipsilateral visual acuity: 0.88 ± 0.04 c/deg; *p* = 0.101 see Fig. 1D), and in Naïve animals (n = 4, controlateral visual acuity: 1.01 ± 0.06 c/deg; ipsilateral visual acuity: 0.97 ± 0.04 c/deg; *p* = 0.200). In full agreement with the behavioral analysis, visual acuity in the formerly amblyopic eye of RUN animals displayed values not significantly different from those of naïve and significantly higher than those of SED rats (p = 0.127 and *p* < 0.001, respectively).

To determine ocular dominance (OD), we measured the contralateral to ipsilateral (C/I) VEP ratio in response to a low spatial frequency grating (0.1 c/deg). In SED rats (n = 9), there was no recovery of OD in the visual cortex contralateral to the formerly deprived eye, which remained significantly lower than in naïve rats (n = 9) (C/I VEP ratio in SED rats = 0.92 ± 0.09 (C = 249.13 ± 37.66 µV, I = 277.56 ± 29.98 µV), in naïve rats 2.76 ± 0.26 (C = 650.38 ± 54.98 µV, I = 257.03 ± 31.77 µV), Fig. 1E, (One-Way ANOVA on ranks, Tukey test, *p* < 0.05). In contrast, RUN rats (n = 9) showed a marked rescue of OD, with a C/I VEP ratio of 2.69 ± 0.36 (C = 572.74 ± 41.33 µV, I = 220.14 ± 24.98 µV). The C/I VEP ratio in RUN rats was significantly higher than in SED rats (One-way ANOVA, on ranks, Tukey test, *p* < 0.05), and was not different from that of naïve rats (n = 9; C\I VEP ratio = 2.76 ± 0.26; *p* > 0.05).

Thus, voluntary physical activity under binocular conditions in the usual living environment induced a marked and persistent recovery of both visual acuity and depth perception in adult amblyopic rats, in correlation with a recovery of a normal visual cortical ocular dominance and of cortical visual acuity.

### Spike analysis of single units

To obtain a detailed characterization of the effects elicited by physical activity on neuronal responses in V1, we performed multichannel electrophysiological recordings^19^ of visual cortical single unit spike activity in non-deprived (naïve, n = 9), long-term deprived exercised (RUN, n = 9), and long-term deprived sedentary (SED, n = 9) rats.

**Figure 2:**
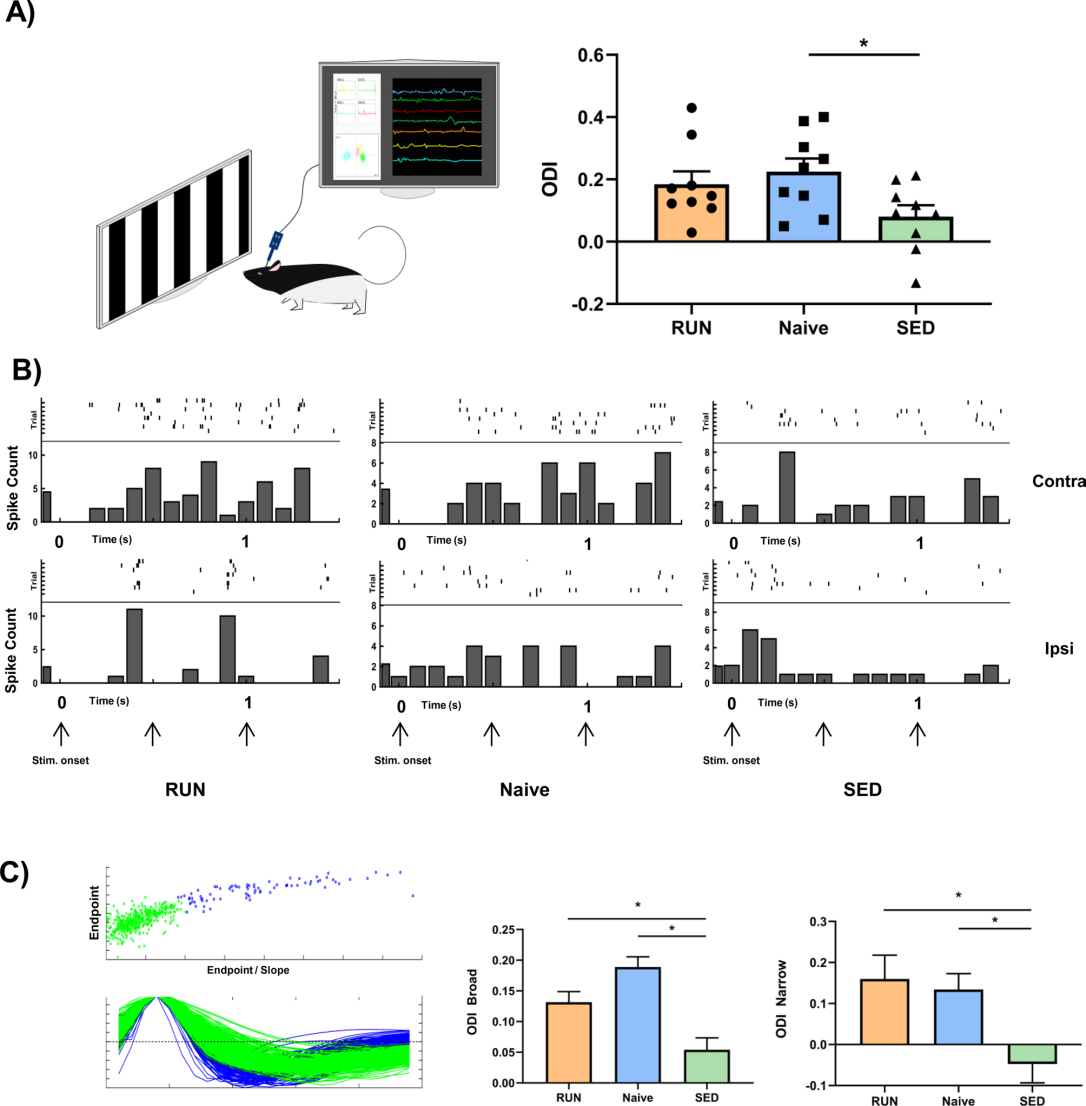
Spike analysis of single units for ocular dominance assessment in long-term deprived rats. (**A**) Assessment of ocular dominance in V1. Plots indicate average and individual ODI values of single animals. While SED rats had a reduced ocular dominance index (ODI) (One-Way ANOVA vs. control, F = 3.429, Holm-Sidak method, *p* < 0.05.), reflecting a shift toward the non-deprived eye, no significant difference was found between naïve and RUN animals (p = 0.1445). (**B**) Raster plots (based on spike counts) for three example neurons, one for each group of animals, in response to stimulation of either eye. (**Inset**) A schematic of the experimental setup for electrophysiological recordings of single units in V1. (**C**) Spike waveforms for all units analyzed, aligned to minimum and normalized by trough depth, demonstrating narrow-spiking (blue; n = 81) and broad-spiking (green; n = 437) units. (**D**) Assessment of ocular dominance in broad (excitatory) and narrow (inhibitory) spiking cells in V1. Long-term deprivation induced an ocular dominance shift in both classes of cells (broad-spiking, SED, n = 137, Naïve, n = 159, One-Way ANOVA, *p* < 0.001; narrow-spiking, SED, n = 23, Naïve, n = 32, One-Way ANOVA, *p* < 0.05). Physical activity induced a full recovery of OD in both classes of cells, and no differences were detectable between naïve and RUN animals (broad-spiking: RUN, n = 141; One-Way ANOVA, *p* = 0.55) (and narrow-spiking cells: RUN, n = 26; One-Way ANOVA, *p* < 0.69). * indicates statistical significance.

**Figure 3:**
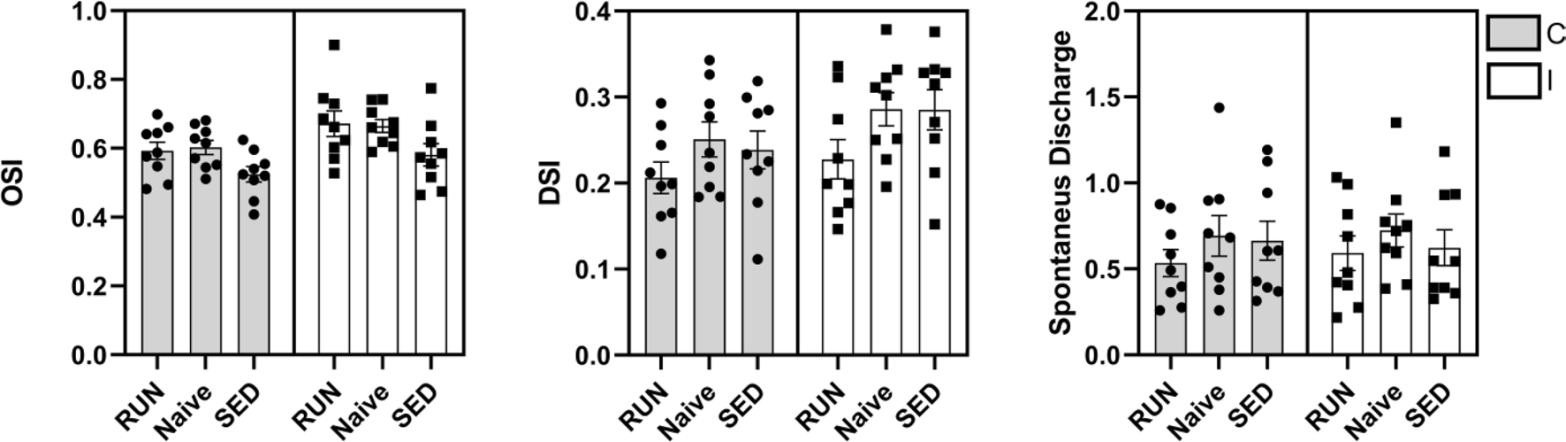
Assessment of direction and orientation selectivity and spontaneous discharge in V1. No difference was found in either the direction selectivity index (DSI), the orientation selectivity index (OSI), or in the spontaneous discharge among the three groups of animals (Two-Way RM ANOVA, Holm-Sidak method, F = 0.150, *p* = 0.861; F = 0.200, *p* = 0.820; F = 0.523, *p* = 0.599; respectively). Error bars indicate s.e.m.; * indicates statistical significance.

Single units were recorded in anesthetized animals in response to drifting sinusoidal gratings that varied in orientation and spatial frequency. We observed a marked reduction of the ocular dominance index (ODI) in SED rats in comparison with naïve animals, reflecting a strong shift of dominance towards the non-deprived eye in the response of V1 single units (One-Way ANOVA vs Control, F = 3.429, DF = 2, Holm-Sidak method, *p* < 0.05.) (Fig. 2A, B). Instead, there was no significant difference in the ODI between naïve and RUN animals (One-Way ANOVA vs Control, Holm-Sidak method, *p* = 0.1445). We found no difference among the three groups of animals in either the orientation selectivity index (OSI), the direction selectivity index (DSI) or in the levels of spontaneous activity (Fig. 3) (Two-Way RM ANOVA, Holm-Sidak method, DF = 2, F = 0.150, *p* = 0.861; F = 0.200, *p* = 0.820; F = 1.384, *p* = 0.270, respectively).

We further investigated ocular dominance separately in putative inhibitory and excitatory neurons, based on spike waveform analysis. Cluster analysis isolated 81 narrow-spiking (inhibitory) and 437 broad-spiking (excitatory) cells (Fig. 2C). In SED rats, both broad and narrow spiking neurons displayed a clear OD shift towards the non-amblyopic eye, when compared with naïve animals (broad-spiking, SED, n = 137, ODI = 0.0541, Naïve, n = 159, ODI = 0.189, One-Way ANOVA, F = 14.505, DF = 2, *p* < 0.001; narrow-spiking, SED, n = 23, ODI = -0.047, Naïve, n = 32, ODI = 0.134, One-Way ANOVA, F = 5.020, DF = 2, *p* < 0.05). On the contrary, physical activity induced a full recovery of OD in both classes of cells, and no differences were detectable between naïve and RUN animals (broad-spiking: RUN, n = 141, ODI = 0.132; One-Way ANOVA, *p* = 0.55) (and narrow-spiking cells: RUN, n = 26, ODI = 0.160; One-Way ANOVA, *p* < 0.69) (Fig. 2D).

### Phenotypical dissection of active GABAergic connections in amblyopia recovery induced by voluntary physical activity

Several papers (reviewed in^13^ have suggested that physical activity modulates the gain of visual cortical responses acting on visual cortical inhibitory interneurons, and, in particular, via disynaptic dishinibition involving two specific subpopulations of cortical GABAergic interneurons. We then addressed the impact of long-term deprivation and exposure to enhanced physical activity on the selective activation of different inhibitory neuronal sub-populations.

**Figure 4:**
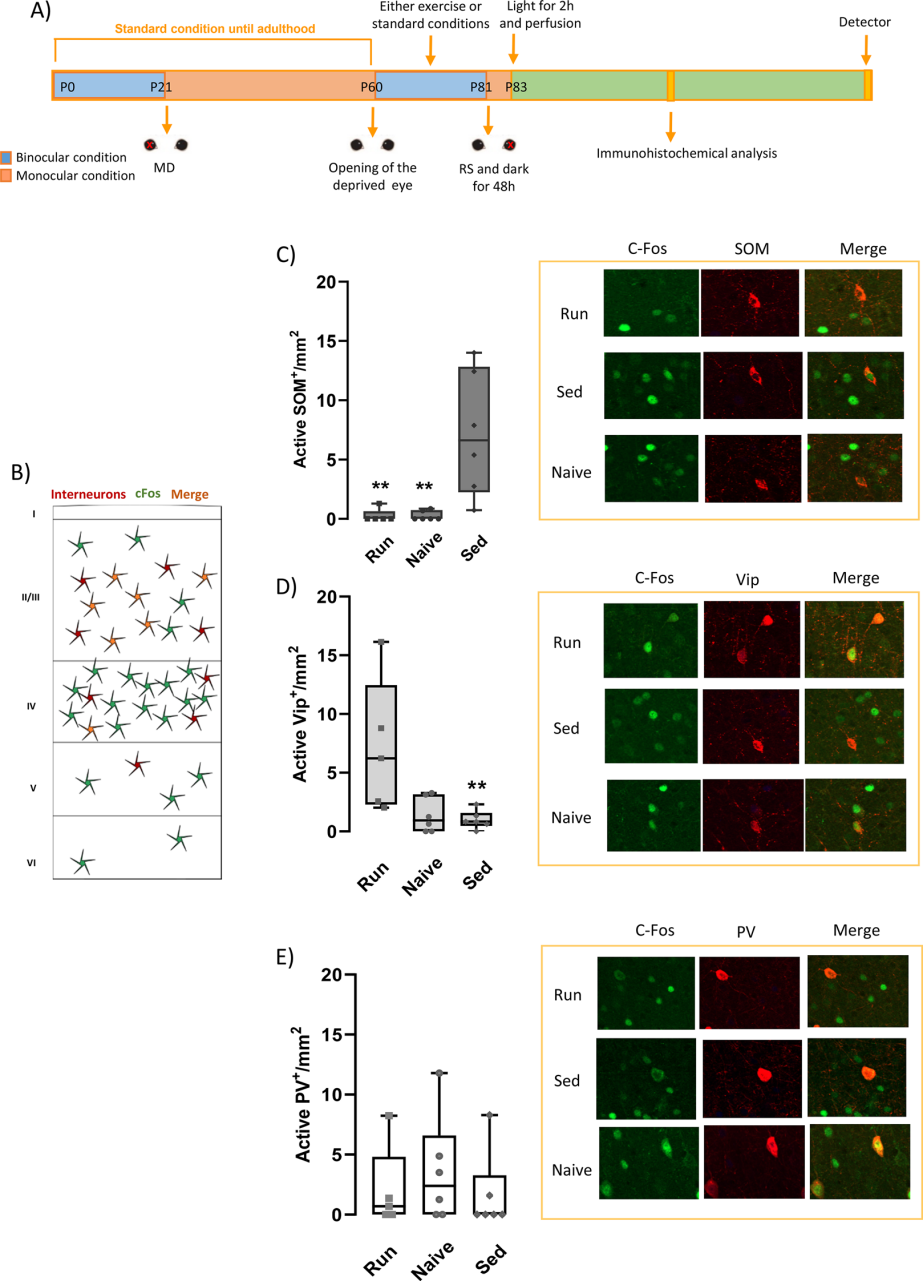
Phenotypical dissection of active GABAergic neurons in amblyopia recovery induced by voluntary physical activity. (**A**) Schematic diagram of the protocol employed for c-fos immunohistochemistry. (**B**) Schematic diagram of interneuron staining in the primary visual cortex . (**C**–**E**) Activation of vasoactive intestinal peptide positive (VIP+, **C**) somatostatin positive (SOM+, **D**), and parvalbumin positive (PARVA+, **E**) interneurons in the primary visual cortex of adult amblyopic animals. The number of SOM+ cells was significantly increased with respect to naïve animals in SED rats, but it returned to normal levels in RUN animals (One-way ANOVA on ranks, Dunn’s Method, q = 2.687 and q = 0.109, respectively). The number of VIP+ cells was significantly increased in the visual cortex of RUN rats with respect to SED animals (One-way ANOVA on ranks, Dunns’ Method, q = 2.453, *p* < 0.05).The number of PARVA+ cells did not change among the three different groups (One-way ANOVA on ranks, q = 0.587). Error bars indicate s.e.m.; * indicates statistical significance. Right panels: representative images of double immunostaining for VIP+/cFos, SOM+/cFos+, and PARVA+/cFos+ cells in the primary visual cortex of RUN, SED and naïve rats. Scale-bar: 12 µm.

To target specific cortical interneurons, we used immunohistochemistry for the activity-dependent marker c-Fos, in conjunction with markers selective for three distinct GABA-ergic subclasses of cells: intestinal peptide positive (VIP+), somatostatin positive (SOM+) and parvalbumin (PARVA+) positive interneurons (Fig. 4B). The analysis was performed in V1 of naïve rats (n = 6) and of RUN and SED rats. To estimate the number of neurons activated by the deprived eye, animals were put in the dark for 2 days at P81, which, in RUN and SED animals, corresponds to the end of the binocular experience with or without physical exercise; at the moment of being put in the dark, the previously not deprived eye was closed, so that, upon light re-exposure, only the previously long-term deprived eye was open (Fig. 4A). Also in naïve animals one eye was closed at dark exposure. In this way, it was possible to evaluate the activation of specific GABAergic interneurons by the long-term deprived eye. We found significant changes in interneuron activation only in layer 2/3. In particular, in SED animals (n = 6), long-term deprivation induced a marked increase in the number of SOM + interneurons activated by the deprived (contralateral) eye compared to naïve animals (SED: 7.200 ± 2.15; naïve: 0.257 ± 0.16) (One-way ANOVA on ranks, Dunn’s Method, q = 2.687, *p* < 0.05) (Fig. 4A). This effect was completely reversed in RUN animals (n = 5), in which the number of SOM + interneurons activated by the deprived eye did not differ from that of naïve rats (RUN: 0.259 ± 0.23) (n = 6) (One-way ANOVA on ranks, Dunn’s Method, q = 0.109, *p* < 0.01) (Fig. 4C). A similar trend was found in layer 5/6 for which, however, differences did not reach statistical significance (SED: 3.064 ± 1.14; RUN: 0.137 ± 0.12; naïve: 0.367 ± 0.24) (One-way ANOVA on ranks, Dunns’ Method, *p* < 0.05 for difference among the three groups; *p* = 0.09 SED vs. naïve; *p* > 0.1 RUN vs. naïve).

In RUN animals, we observed an increment in the number of active VIP + interneurons in comparison with SED rats in layers 2/3 (RUN: 7.149 ± 2.56; SED = 0.852 ± 0.31; naïve: 1.375 ± 0.60) (Fig. 3b) (One-way ANOVA on ranks, Dunns’ Method, q = 2.453, *p* < 0.05) (Fig. 4D). No difference was found in the number of VIP + active interneurons among naïve, RUN and SED animals in layers 5/6 (One-way ANOVA on ranks, Dunns’ Method, *p* = 0.948) (SED: 3.064 ± 1.14; RUN: 0.137 ± 0.12; naïve: 0.367 ± 0.24) (SED: 0.080 ± 0.08; RUN: 0.313 ± 0.28; naïve: 0.162 ± 0.16).

No difference was found in the number of active PARVA+ interneurons among naïve, RUN and SED animals (One-way ANOVA on ranks, q = 0.587) (Fig. 4E), in agreement with previous results^23^.

In conclusion, amblyopia was associated with an increased activation of SOM+ interneurons and a decreased activation of VIP+ interneurons by the deprived eye; physical activity fully reverted this effect, with an increase in the activation of VIP+ interneurons and a decrease in the activation of SOM+.

## Discussion

The findings reported in the present study show that voluntary physical activity performed under binocular conditions in the usual visual environment promotes a marked and long lasting recovery of visual functions in adult amblyopic rats. Both visual acuity and depth perception recovered, reaching the same levels of rats with typical development, and the recovery remained stable for at least 6 months. This is a rather long period of recovery stability in terms of rat life span, which, translated in terms of humans, would correspond to several years.

Most papers in the large literature on amblyopia recovery in animal models have employed reverse-occluded animals^5,24–26^. Rescue of visual functions was here achieved in rats attaining physical activity under conditions of normal binocular sight. This is central in terms of possible translational applications to human patients, in which protocols of reverse eye occlusion are of very limited interest due to potentially relevant and intrinsic risks^15,27^. While running coupled with short-term occlusion of the amblyopic eye has been reported to elicit substantial visual function recovery in adult anisometropic amblyopes^11^, the present study suggests the possibility of a rescue of visual functions in amblyopic rats even in the absence of eye patching. Actually, in the Lunghi et al. work^11^, subjects spent the vast majority of their time with unrestricted binocular vision, similarly to the condition we tested in amblyopic rats, and short-term eye patching was used only during the periods of physical activity. In this condition, short-term eye patching is suggested to evoke a form of homeostatic plasticity in the adult visual cortex, with time-restricted physical exercise acting as a specific boost for it, and the subsequent binocular vision driving recovery of both visual acuity and stereopsis. In the present study, unrestricted physical activity is suggested to act as a general boost for visual cortical plasticity and activity, with concomitant binocular vision driving recovery of visual acuity and stereopsis. We do not know, at present, whether physical exercise per se, not coupled with amblyopic eye patching, might promote some recovery of vision also in amblyopic humans.

Increasing the potential exploitability of physical exercise in human amblyopia recovery are the data showing that physically exercised rats displayed a remarkable recovery of visual depth perception abilities. Deficits of stereopsis are widely considered the most disturbing impediment in normal everyday life for amblyopic subjects^14,15^, calling for procedures able to counteract them, independently on visual acuity^17,28^.

It has been previously shown that 3 weeks of exposure to environmentally enriched conditions under binocular conditions rescue the ocular dominance and the binocular matching deficits caused by an early visual deprivation during the CP in mice^29^, and a recovery of deprived eye responses was also reported in head-fixed animals in which binocular visual stimulation with specific visual stimuli was paired with running on a treadmill^9^. Here, rescue of the OD deficit in exercising animals was for the first time linked to recovery not only of visual acuity (see also^12^, but also of visual depth perception abilities, directly assessed at the behavioural level. The Levine et al.^29^ paper also offers an interesting answer to the criticism, sometimes moved to rodent models of human amblyopia, that monocular deprivation in rats, given that it reduces the strong contralateral bias present in rodents, increases the number of binocular neurons in the primary visual cortex contralateral to the deprived eye, and this is not easily reconciled with a reduced binocular vision (depth perception). The Levine paper strongly suggests that the sheer number of binocular neurons in V1 is not sufficient to sustain a good binocular vision: it is a necessary, but not a sufficient factor. In monocularly deprived mice the binocular matching process is completely disrupted, resulting in binocular visual cortical neurons displaying different optimal orientations in response to the left or the right eye, with foreseeable consequences for binocular vision. Environmental enrichment, which rescues both ocular dominance and binocular matching^12,29^, also rescues depth perception^17^. In the Kaneko and Stryker paper^9^, physical exercise in head fixed animals exposed to specific visual stimuli for 4 h per day led to recovery of normal levels of response to the deprived eye for excitatory neurons, while narrow-spiking (inhibitory) neurons remained less active. We found that ocular dominance recovered both for excitatory and inhibitory neurons in physically exercised rats. This may be due to the different manner of physical exercise (free running vs head fixed) and to the ecological, varied, prolonged visual stimulation animals experience while running on the wheel in their cage.

It is interesting to notice that, in our model, animals were allowed to exert voluntary physical exercise at any moment of the day. When running is performed during the light phase of the day, animals receive natural visual stimulation from the surroundings, coupled with strong visual stimulation arising from the running wheel; this is likely to generate a condition of much more sensory richness than that associated with the condition of specific visual stimuli coupled with the exercise bouts reported in the paper of Kaneko et al.^9^. On the other hand, it has been demonstrated that running in the absence of any visual stimulation can also strongly activate the disinhibitory Vip-SST-Pyr network in the visual cortex^30^; thus, activation of this neural circuit may have been elicited, in our paradigm, also when rats performed physical exercise during the dark phase of the day.

The water maze task we used to assess behavioral visual acuity did not per se result in any amount of visual function recovery in the control group of SED rats. This is in line with previous work showing that visual acuity assessment in this task does not ameliorate visual discrimination performance, even when it is repeated several times^18^, possibly as result of the stressful component of forced physical exercised intrinsic to the task^31^. Conversely, a modified version of the same task can lead to marked visual function recovery in adult amblyopic rats when used to perform visual perceptual learning, a strong and highly targeted visual training that is able to robustly enhance visual discrimination performances^32,33^. We previously showed that while this form of visual perceptual learning is linked to LTP-like changes in visual cortex circuitries, this effect is not seen in control animals lacking the incremental component of the visual training task^34^.

When focusing on GABAergic connections, c-fos immunohistochemistry revealed a differential regulation in the activity of distinct sub-populations of interneurons. Specifically, amblyopia resulted in increased numbers of active SOM+ interneurons, without any detectable effect on either VIP+ or PARVA+ cells. In contrast, physical exercise was associated with both a specific increase of active VIP+ cells, and a restoration to basal numbers of active SOM+ interneurons. These results fit well with the framework of a recent model put forward by Stryker and coll.^13^. In this model, running in restrained animals enhances visual cortical activity in non-deprived mice, and favor recovery from amblyopia in deprived subjects, via a disynaptic disinhibitory circuit whereby activation of VIP+ interneurons increases inhibition of SOM+ cells in the visual cortex, thus disinhibiting pyramidal neurons. Activation of this circuit was previously reported only in head-fixed mice during individual bouts of high locomotor activity, and was never associated with direct assessment of visual recovery in amblyopia animals, with a functional analysis in line with clinical analysis in human patients. Our data show for the first time that the same disinhibiting circuit is also active in rats in which a fully spontaneous running behavior takes place, and that this activation persists well past the end of the individual bouts of physical activity. Indeed, c-fos immunohistochemistry was performed following a 48 h protocol of dark and light exposure in the absence of any running wheel, at the end of the physical exercise three-week regimen.

No effect was instead found at the level of global activation of PARVA+ cells, which displayed a low responsiveness to the effects of long-term MD deprivation, in agreement with previous work^23^. However, spike sorting indicated that the specific change of ocular preference in response to MD was similar for both narrow spiking, putative inhibitory PARVA+ neurons, and for broad spiking, putative excitatory neurons, as also previously reported in juvenile rats during the CP^35^. Thus, the balance between excitation and inhibition in the visual cortex appeared to be preserved after long-term MD and after recovery from amblyopia induced by physical activity. This might be essential to ensure a proper encoding of visual information, independently on the OD shift.

Running in head-fixed mice was previously reported to depend on activation of V1 cholinergic afferents from the midbrain locomotor region^36^. In addition to this link between physical activity and V1 recovery, physical activity might also have elicited a rescue of visual functions acting on peripheral factors capable to promote brain epigenetic changes, finally controlling the action of specific genes involved in V1 plasticity, like the *bdnf* gene^20,37,38^. Indeed, physical activity enhances the expression of BDNF through the action of the ketone body β-hydroxybutyrate, and BDNF was recently shown to play a remarkable and specific action in eliciting recovery from amblyopia in adult amblyopic rats^18^.

A point that was not addressed in the present study was whether the changes in visual perception (visual acuity and depth perception), and those at the level of OD and activity of GABAergic interneurons elicited by motor activity were correlated among each other’s. Since behavioral, electrophysiological and immunoistochemistry were performed on different cohorts of animals, it was not possible to perform this type of analysis. Future research may specifically focus on this issue.

In conclusion, we have demonstrated that spontaneous physical activity (free running) applied in unrestricted conditions of ordinary binocular vision effectively rescues visual functions in adult amblyopia and also rescues visual responses in the primary visual cortex . These findings could have a bearing in orienting clinical research in the field of amblyopia therapy.
